# Supplementary material for: Are carbon emissions trading and green financial instruments synergistic? -Comprehensive quantitative research based on content analysis
Source: PLoS One. 2024 Mar 7;19(3):e0298601. doi: 10.1371/journal.pone.0298601 (PMC10919601; doi:10.1371/journal.pone.0298601)
Supplement: S1 Data — (ZIP) [file pone.0298601.s001.zip › Data/地级市Z_F工作报告.zip!/║■▒▒/╛ú├┼╩╨/└┤╫╘╣1⁄2╓┌║┼í╛┬φ┐╦╩2╛▌═°í┐_2005.docx]

各位代表：

　　现在，我代表市人民政府向大会作工作报告，请予审议，并请各位政协委员和列席人员提出意见。

　　一、2004年工作回顾

　　过去的一年，是我市近几年来经济发展速度最快、效益最好、人民群众得到实惠最多的一年。在省委、省政府和市委的正确领导下，在市人大、市政协的监督与支持下，市政府团结依靠全市人民，树立和落实科学发展观，求真务实，改革创新，超额完成了市六届人大二次会议确定的主要预期目标。

　　----经济全面快速发展。全市生产总值完成379.46亿元，增长11.5％，其中一、二、三产业分别增长6.3％、15.5％和9.3％。投资、消费和出口持续增长。完成全社会固定资产投资86.13亿元，增长21.4％，创八年来最高水平。社会消费品零售总额120.43亿元，增长13.1％。进出口总额首次突破1亿美元，其中出口9063万美元，增长35.6％。

　　----财政金融平稳运行。完成地域财政收入22.88亿元，按可比口径增长16.4％；地方一般预算收入9.29亿元，按可比口径增长20.6％。金融机构存、贷款余额分别比年初增加27.73亿元和3.96亿元。

　　----城乡居民收入大幅增加。城镇居民人均可支配收入7749元，增长9.8％；农民人均纯收入3629元，增加504元，增长16.1％，增幅是多年来最大的一年。

　　----就业工作成效明显。新增就业25988人，超预期目标29.9％；城镇登记失业率为4.2％。

　　一年来，我们高度重视并认真做好打基础、管长远和根本性的工作。倡导科学务实的工作作风，遵循市场经济的基本规律，把主要精力放在调整结构、招商引资、培植发展后劲上，把工作重点放在体制创新、制度建设和环境改善上，把有限的财力、物力更多地用在解决关系人民群众切身利益的突出问题上，努力实现经济社会的可持续发展。

　　（一）坚持“兴工富市”，改善经济结构。围绕增强综合竞争力，重视经济理论学习与发展战略研究，制定并实施《荆门市新型工业化规划纲要》。努力培育和发展支柱产业、优势行业、龙头企业和名牌产品，荆门石化、洋丰集团、京山轻机年销售收入分别达到110亿元、15亿元和12亿元。全市新增亿元工业企业9家；荆门高新区和京山、钟祥工业园区发展势头强劲。加强经济运行调节，缓解煤电油运制约，提高工业经济运行效益，全市规模以上工业完成增加值72亿元，增长23.1％，利润总额增长2.32倍；工业经济综合效益指数达142.9，比上年提高26.4个百分点。抓好重点项目建设与服务，荆门热电厂三期扩建工程已进入国家核准程序；荆门石化原油加工能力在2至3年内将扩大到600万吨；世界著名啤酒企业英博集团与金龙泉啤酒集团顺利合资；世界500强的中粮集团在钟祥投资办厂；金龙非织造布、永兴水泥、宝源中高密度板、北山抽水蓄能电站等一批投资过亿元的项目建设进展顺利。努力提高服务业在国民经济中的比重，旅游经济发展创历史新高，房地产、金融保险、交通运输、邮电通信、信息咨询和社区服务等服务业进一步发展。我市再次被授予“全省A级金融信用城市”称号。

　　（二）坚持落实政策，全面发展农村经济。积极宣传、认真落实党的农村政策，进一步调动农民生产积极性，农业和农村经济呈现多年少有的好形势。粮食总产量达201万吨，增产32.8万吨，棉花、油料、林果、畜禽、水产品、食用菌等产量全面增长。沙洋县、钟祥市获“全国粮食生产先进县”称号。深入推进农业产业化经营，全市新增优质农产品基地20万亩、畜禽养殖小区21个、“三品”（无公害食品、绿色食品、有机食品）生产面积52.1万亩、规模以上龙头企业16家，日月油脂、洪森实业被列入国家级龙头企业。京山桥米获国家原产地域产品保护，实现了我省零的突破。积极推进农村税费改革、乡镇综合配套改革、国有农场综合改革，农民负担明显减轻，全市农民负担总额比上年下降43.9％，亩平负担下降44.4％。乡镇企业增加值、营业收入和利润总额的增幅均居全省前列。

　　（三）坚持深化改革，扩大对外开放。荆襄化工（集团）公司下放荆门后，改革脱困实现历史性突破，集团公司改制、剥离社会职能及荆源公司破产已全面完成，大峪口项目联动试车在即。宏图飞机制造厂平稳进入破产程序，凯龙集团、东光集团引进战略投资者的工作扎实推进，市直改制企业遗留问题得到高度重视并在逐步解决。落实促进非公有制经济发展的各项政策措施，全市个体工商户、私营企业分别新增13240户和821户。积极推进国有资产监管体制改革，探索建立集经营性、非经营性、公益性与资源性资产为一体，实施全方位监控的国有资产监管体系。顺利完成政府机构改革，稳步开展事业单位人事制度改革。财政管理体制、投融资体制和国土资源管理体制改革取得新进展。对外开放不断扩大，全市新增外资企业21家，全年吸收外商投资6825万美元，增长20.7％。

　　（四）坚持统筹兼顾，促进协调发展。加快生态园林城市建设，筹资3.3亿元，配套南城区基础设施，完善老城区道路、水、电、气等公共服务设施。象山大道南端建成通车。健全城市管理职能，改善城市市容与交通监管装备，市容环境与道路交通秩序综合整治成效明显。重视并加强社区建设，推进各项职能逐步到位。城南新区综合整治初见成效，人居环境明显改善。积极推进科技、教育、文化、卫生、体育、人口和计划生育等各项社会事业发展。高新技术产业增加值比上年增长25.4％。教育事业稳步发展，九年义务教育完成率达到92.4％，荆门职院与沙洋师专合并及升本工作进展顺利。社会科学、文学艺术、新闻出版、广播电视事业繁荣活跃。成功举办庆祝第五个中国记者节暨新闻奖文艺晚会，社会文化获全国“群星奖”。市县疾病预防控制和医疗救助体系不断完善，农村新型合作医疗试点工作启动；我市荣获“全国无偿献血先进城市”称号。我市运动员何军权在雅典残奥会上勇夺4枚金牌。人口自然增长率为1.6‰。大力整顿土地市场，严格保护耕地。加强生态建设和环境保护，漳河水源保护工作全面展开，青山水泥厂“退二进三”工作取得突破性进展。森林覆盖率达到35.7％。高度重视并加强安全生产工作，实现安全控制指标全面下降。加强社会治安综合治理，积极预防和依法处置群体性事件，维护了社会稳定。国防动员、民兵预备役工作不断加强，统计、物价、工商、质量技术监督、食品药品监督、烟草专卖、民族、宗教、对台、外事、侨务、气象、人防、档案、方志、保密、信访、老龄、残疾人和住房公积金管理等工作取得新成绩。

　　（五）坚持依法行政，建设服务型政府。全面贯彻实施行政许可法，废止市级规范性文件93件，占19.9％；取消和调整62项市级政府部门审批事项，占8.7％。坚持向市人大及其常委会报告工作制度，重大决策征求市政协和民主党派、工商联、无党派人士及人民团体意见。全年办结办复人大议案、代表建议和政协提案394件。按照“依法行政、规范管理、权责明晰、纪律严明”的要求，完善政府工作规则和流程，努力提高行政效率。成立市行政服务管理委员会，加强和完善社会管理和公共服务。积极推行政府信息公开，以“中国.荆门”网站的建设与应用带动电子政务发展。强化行政监察和审计监督，积极开展行风评议，加强廉政文化建设，源头治腐取得成效。

　　（六）坚持以人为本，着力解决关系群众利益的实际问题。将符合条件的8.8万人全部纳入低保范围，确保国有企业下岗职工基本生活费、离退休人员基本养老金、失业人员失业保险金和低保对象低保金按时足额发放。全市养老、失业、医疗、工伤、生育等参保人数达79.6万人次，发放社保资金4.06亿元。清欠建筑领域工程款1.19亿元，偿付拖欠农民工工资1417万元，清退征地农民补偿费5612万元，国家公职人员偿还农村信用社贷款本息5555万元。市政府承诺的十件实事顺利完成：全市共有17038名下岗人员实现再就业，一家两人以上下岗失业的家庭实现再就业1925人，超额完成承诺目标；新建通村四级公路483公里，超额完成承诺目标；南城区医院如期建成并投入使用；对所有晚期血吸虫病人实行免费治疗，建立起艾滋病防治机制；资助2.4万名贫困中小学生入学，改造农村中小学D级危房4.6万平方米，超额完成承诺目标；城区大气质量达到国家二级以上标准的天数占85.1％，漳河饮用水源水质达标率为100％；柴湖扶贫脱困项目基本完成年度工作目标；积极服务中央、省属企业改革发展；解决了农村6227人饮用水问题；救助农村特困人口20551人，农村“五保户”集中供养率达82％，超额完成承诺目标。

　　上述成绩是全市人民团结奋斗、辛勤劳动的结果。在此，我代表市政府向全市人民，向驻荆部队、武警官兵和公安干警，向各民主党派、工商联、无党派人士和人民团体，向所有关心、支持和参与荆门建设的各界朋友表示衷心的感谢并致以崇高的敬意！

　　在总结成绩的同时，我们也清醒地看到，经济社会生活中仍然存在着一些突出矛盾和问题。主要是：农村经济发展基础脆弱，农民增收的长效机制尚未形成；经济运行质量和效益不高，高投入、高消耗、高污染与低产出的增长方式仍然占据主导，加剧了资金、土地和煤电油运等要素资源的紧缺，导致财政状况与经济社会发展要求的不相适应；结构调整任务艰巨，优势骨干企业不多，高新技术产业规模不大，非公有制经济和开放型经济发展不够，县域经济有待壮大；就业再就业任务繁重，社会保障体系有待进一步健全，部分群众生活比较困难，安全生产形势依然严峻；一些部门和单位服务意识不强、行政效率较低，形式主义、官僚主义、腐败现象依然存在。之所以产生这些矛盾和问题，源于制约经济发展的体制性、机制性与结构性障碍尚未根本突破，反映出现存的管理体制、运行机制与市场经济发展要求的差距，反映出长期计划经济形成的观念、思路和方法与坚持科学发展观要求的差距，反映出传统的经济增长方式与坚持走新型工业化道路要求的差距，反映出经济发展的成效与人民群众迅速增长的物质文化需求的差距。对这些问题，我们将高度重视并认真有效地加以解决。

　　二、2005年政府工作的总体要求和预期目标

　　今年政府工作总体要求是：以邓小平理论和“三个代表”重要思想为指导，全面贯彻党的十六大、十六届三中、四中全会精神，以科学发展观统揽全局，以加快经济结构调整、促进产业升级、转变增长方式为重点，进一步深化改革、扩大开放、聚合资源、优化配置，继续实施“兴工富市”战略，统筹城乡发展，完善社会保障体系，不断扩大就业再就业，着力解决关系人民群众利益的突出矛盾和问题，保持社会和谐稳定，促进经济社会全面协调可持续发展。全市经济社会发展的主要预期目标是：生产总值增长11％，其中一、二、三产业分别增长5％、14％、10％；规模以上工业增加值增长18％以上；全社会固定资产投资增长18％；社会消费品零售总额增长12％；外贸出口与外商投资均增长20％左右；地域财政收入可比增长14％，地方一般预算收入可比增长18％；城镇居民人均可支配收入增长6％，农民人均纯收入增长5％；城镇新增就业2.4万人，城镇登记失业率控制在4.4％以内；人口自然增长率控制在3‰以内。

　　确定以上预期目标，主要是考虑与我市发展位势和比较优势相一致，与全市上下加快发展的愿望相符合，与完成“十五”计划目标相衔接。从宏观经济环境看，全球产业结构调整与重新分工进程加快，国际资本看好我国经济的持续快速发展，沿海产业正在向中西部梯度转移，国内非公有制经济加速发展，资本市场日趋活跃；国家将进一步加大对农业、农村的政策扶持力度。从我市经济发展看，“兴工富市”战略实施近三年来，一批带动力强的工业项目陆续竣工投产，新的经济增长点逐步形成；日益凸现的交通区位优势、得天独厚的农业生态资源、储藏丰富的矿产资源和前景广阔的旅游资源，正不断聚合成新的发展优势和竞争力。这些都为我们发挥比较优势、挖掘发展潜力、调整经济结构提供了新的机遇。

　　三、促进经济持续快速协调健康发展

　　发展是落实科学发展观的核心。加快发展必须突出结构调整这条主线，把握增长方式转变这一重点，抓住改革开放这个关键。

　　（一）坚持走新型工业化道路，努力培植新的经济增长点

　　推进产业升级。加快实施新型工业化规划纲要，广泛运用信息、节能、新型环保等高新技术和先进适用技术改造提升传统产业，推进重点企业和重点行业的技术改造，加快新产品开发。做强做大石油化工、磷化工和能源工业，改造提升机械冶金、建材等传统制造业，延伸壮大食品工业、纺织工业及农副产品加工业，积极培育新医药、电子信息、新材料等高新技术产业。

　　做强做大骨干企业。全力服务荆门石化、荆门热电厂、葛洲坝水泥厂等中央、省属企业的改革发展。大力扶持金龙泉集团、洋丰集团、百科药业、京山轻机、凯龙集团、宝源集团、东光集团、荆玻集团、金龙非织造布等企业的发展，培育核心竞争力。继续实施“亿元工程”和精品名牌战略，发展成长型、科技型中小企业，力争用2至3年时间，在全市培育1家年销售收入过150亿元、10家过10亿元、80家过亿元的工业企业。

　　加快园区和项目建设。努力把荆门高新区建设成为高新技术产业的聚集区、现代制造业的密集区和兴工富市的示范区，把207国道钟祥胡集段和东宝段建成磷化工和新型建材工业走廊。实施产业聚集和产业配套，加快京山、钟祥、沙洋等工业园区建设。加大工业有效投入，重点服务荆门石化扩能改造、永兴水泥、中粮油脂加工、宝源中高密度板、庆喜维新纺织等续建项目和荆门热电厂三期扩建、北山抽水蓄能电站、洋丰合成氨、嘉信超细纤维造纸、华贝硫酸钾等新开工项目建设。加强和改善对经济运行的调节，做好煤电油运等要素资源的协调与科学调配。盘活存量土地，提高用地效率。

　　（二）以农民增收为中心，加快农业和农村经济结构调整

　　提高农业综合生产能力。严格保护耕地，推进科教兴农。广泛开展“农业科技推广年”活动，认真做好农业技术推广、农业机械化和科技入户工作，突出抓好优质高产良种、高产高效模式、无公害农产品标准化生产、测土配方施肥、动植物疫病防治及避灾技术等实用技术的推广应用。着力建设京山、钟祥、沙洋等国家优质粮产业工程基地和高产农田，积极申报国家大型商品粮基地。加强农田水利基本建设，完善农业用水机制，抓好堤防整治、病险水库除险加固和渠系维修配套，加强农作物病虫害和动物疫病防治体系建设，提高农业抵御自然灾害能力。做好气象预警预报工作。

　　培育特色经济。继续加强八大优势产业和八大特色产品基地建设，发展专业镇、村，扩建新建畜禽养殖小区，重视加快城郊标准化蔬菜基地建设，加强速生丰产林基地和沿国道、省道花卉苗木基地以及高效经济林基地建设，努力形成各具特色、优势明显、竞争力强的产业带。

　　推进农业产业化。充分发挥农业比较优势，坚持以工业化理念推进农业产业化。抓住市场化取向、区域化布局、专业化种养、标准化生产四个关键环节，创新农业产业化经营机制。加强对日月油脂、洪森实业、国宝桥米、宝源集团等重点龙头企业的扶持，完善龙头企业与基地、农户的利益分配关系，在深加工过程中提高农副产品附加值。依托龙头企业、基层供销社、经销大户、经纪人等，积极发展多种形式的农村合作经济组织，广泛开展技术、信息、流通等服务。大力发展打工经济，加快转移农村劳动力。

　　贯彻落实各项政策。坚持“多予、少取、放活”的方针，认真落实免征农业税、取消农业特产税、对种粮农民实行直接补贴、良种补贴和农机具购置补贴等各项惠农政策。增加市、县两级对农业的投入。加强柴湖、漳河库区等贫困地区及革命老区的扶贫开发，搞好结对扶贫、对口扶贫和搬迁扶贫。

　　（三）大力发展现代服务业，积极扩大消费需求

　　加快发展旅游业，培育旅游投资开发主体，坚持以市场方式整合旅游资源；加强以明显陵、大洪山、漳河为代表的风景名胜区等旅游资源的系列开发，规划建设中心城区旅游圈，打造中国优秀旅游城市整体形象。加强房地产开发管理，积极搞活住房市场，促进房地产业健康发展。完善住房公积金管理制度。扩大廉租住房受益面。大力发展现代物流业，推行连锁经营、物流配送、现代批发等新型业态，积极发展电子商务及信息、保险、咨询等现代服务业。努力改善消费环境，培植住房、汽车、通信、旅游、教育、文化等消费热点，拓展消费领域。

　　（四）进一步深化改革，增强经济发展活力

　　深化国资与国企改革，促进非公有制经济发展。完善国有资产管理体制和监管方式，落实经营性国有资产的保值增值责任制，防止国有资产流失。按照建立现代企业制度的要求，加快建立和完善法人治理结构，形成科学有效的企业内部决策、执行与监督机制。服务好荆门石化等中央、省属企业的主辅分离，继续做好荆襄公司的改革脱困与新增长点的培育，搞好宏图飞机制造厂破产重组，推进凯龙集团、东光集团等企业的资产重组。按照建立现代产权制度的要求，通过规范国有资产、产权与土地交易，调整国有经济布局，多渠道筹措改制资金，妥善解决国企改制遗留问题。加快供水、燃气、公交等公用企事业单位改革、改组；积极探索学校、医院等事业单位改革，鼓励非义务教育由品牌优势向竞争优势的转换。认真贯彻落实《国务院关于鼓励支持和引导个体私营等非公有制经济发展的若干意见》，消除制约非公有制经济发展的一切障碍，鼓励民间资本投向“非禁即入”领域，维护各种所有制企业的国民待遇。

　　深化财政和投资体制改革。完善预算编制制度，硬化预算约束；完善国库集中收付制度，推行零余额账户管理；完善政府采购操作规程和运行机制；严格落实收支两条线制度，实行收缴分离、罚缴分离、收支脱钩。整合和优化配置财政资源，加大对社会公共需要和社会保障投入。建立农村义务教育最低经费保障和县级政法机关最低经费保障制度，杜绝教育乱收费和公检法创收。加强财政资金特别是专项资金的监督管理和审计，严肃财经纪律，防范财政风险。依法治税理财，积极培育税源，强化税收征管，努力提高财政收入占生产总值和工商税收占财政收入两个比重，实现财税增长与经济增长协调发展。加快投资体制改革，按照“谁投资、谁决策、谁受益、谁承担风险”的原则，规范投资行为，强化企业的投资主体地位。支持金融改革与发展，综合运用各种金融工具，改善金融服务，优化信贷结构，加大金融对经济发展的支持力度。

　　深化农村改革。继续深化农村税费改革，强化涉农收费治理，严格规范“一事一议”的筹资筹劳制度，积极探索农村公益事业发展的新途径。推广京山县精简机构、裁减冗员的经验，深入推进乡镇综合配套改革。依法完善农村土地二轮延包，稳定土地承包关系，规范土地流转。积极慎重化解村级债务，力争今年村级债务再化解10％以上。完善国有农场综合改革。

　　（五）优化经济发展环境，加快对外开放

　　努力营造“开放荆门”的发展环境。进一步优化行政环境、法制环境、市场环境、信用环境和舆论环境，增强区域综合竞争力。强化市场监管，继续整顿和规范市场经济秩序，严厉打击制假售假、商业欺诈等违法犯罪行为。加快完善社会信用体系，加强失信惩戒，巩固金融信用县市创建成果。严肃查处破坏经济发展环境的案件，切实做到公正执法、高效行政、诚信经营。强化舆论宣传，形成“人人都是投资环境、个个都是荆门形象”的社会氛围。

　　提高招商引资质量和水平。引导外资投向高新技术产业、农业产业化经营、现代服务业和基础设施建设，力争在引进世界500强和国内外知名企业方面有新突破。不断创新招商引资方式，积极开展园区招商，激活项目招商，推行中介招商和专业化招商等，力争全年引进外资超过1亿美元。

　　着力转变对外贸易增长方式。调整优化出口商品结构，提高技术含量，扩大农产品及其加工品、机电产品、医药产品出口。支持京山轻机、永祥箱包等企业增加出口，积极培育金龙非织造布、鑫丰新材料、新美香食品、兴利食品、山缘香菇、顺昌蜂业等新的出口增长点，力争全年外贸出口超过1亿美元。培育多元化外贸经营主体。加快筹建海关荆门办事处。鼓励有条件的企业到境外投资办厂，对外开展工程承包、劳务输出。

　　四、努力构建和谐荆门

　　坚持以人为本，构建民主法治、公平正义、诚信友爱、充满活力、安定有序、人与自然和谐相处的社会，是落实科学发展观的本质。

　　（一）加大就业和社会保障力度，努力改善人民生活

　　千方百计增加就业。强化就业再就业目标责任制，认真落实税费减免、社保补贴、小额贷款等政策，解决好困难群体再就业问题。统筹城乡就业，将农村剩余劳动力转移就业纳入公共就业服务体系，为农民外出务工提供信息、培训、劳务派遣和维权等“一条龙”服务。今年农村剩余劳动力转移培训要达到1.2万人，力争新转移3万人以上。加强劳动保障监察，强化劳动合同管理，规范企业用工行为。

　　健全社会保障体系。以提高非公有制经济从业人员、灵活就业人员和破产改制企业职工的参保率为重点，进一步扩大社会保险覆盖面，力争今年社保扩面5.4万人以上。依法加大社会保险费征缴力度，完善事业单位养老、医疗制度改革。积极探索特困人员、失地农民及农民工参加社会保险的途径和方法。

　　切实保障困难群体基本生活。严格执行城市居民最低生活保障和农村特困人口救助政策，实行应保尽保、应救尽救。积极做好社会福利、社会救济工作，努力改善群众生活，让改革发展成果惠及全市人民。重点帮助失业职工、失地农民、拆迁居民和贫困家庭解决生产生活困难，逐步解决看病难、子女上学难等问题。倡导各类社会组织和社会成员开展经常性的扶贫济困送温暖活动。严禁拖欠农民工工资和征地农民的补偿费。

　　（二）加强精神文明建设，全面发展社会各项事业

　　进一步实施科教兴市和人才强市战略。推进与华中科技大学、华中农业大学、武汉理工大学、中科院武汉分院等（院）校的合作，积极促进产学研结合。加快企业技术中心建设，鼓励企业加大研发投入，提高自主创新能力，培育技术创新主体。进一步落实党政主要领导科技进步目标责任制。加强信息技术在经济社会各领域的应用。合理配置教育资源，统筹城乡各级各类教育协调发展。巩固和完善以县为主的农村义务教育管理制度，高度重视义务教育特别是农村义务教育的投入，继续做好扶贫助学工作。大力发展职业教育，稳步发展成人教育和高等教育，做好本科院校的创办工作。扩大和规范社会力量办学。加强教师队伍建设，提高教育质量。高度重视培养、引进、重用、优待各类创新人才，实施“113”人才和高级技能人才发展计划，盘活用好地域人才存量。

　　加快发展文化、卫生、体育等社会各项事业。深化文化体制改革，积极探索以市场运作方式繁荣公益性文化事业的新途径，培育文化精品、壮大文化产业。促进社会科学、文学艺术、新闻出版等事业发展。努力扩大广播电视覆盖面，加快有线电视数字化步伐。搞好文物保护，加强文化市场和互联网管理。做好“八艺节”荆门分会场筹备工作。加快农村文化建设，丰富群众文化生活。完善公共卫生体系，落实艾滋病、血吸虫病等重大传染病和各类职业病防治措施，提高公共卫生保障水平。深化城镇医疗体制改革，积极推进新型农村合作医疗，提高农村医疗卫生服务能力。广泛开展群众性体育活动，发展竞技体育，备战省运会。继续贯彻实施《公民道德实施纲要》，加强社会公德、职业道德和家庭美德建设，深入开展文明社区、文明村镇、文明行业、文明单位、文明家庭创建活动和“做文明公民、建诚信荆门”的道德实践活动，积极倡导科学、健康、文明的生活方式。抓好未成年人的思想道德教育、大学生思想政治教育和全民普法宣传教育。依法保障妇女、未成年人和老年人合法权益，关心和支持残疾人事业。深入开展国防教育，完善国防动员体制，积极做好民兵预备役、“双拥”工作、优抚安置和人防工作，关心和支持驻荆解放军和武警部队的现代化建设，巩固军政军民团结。进一步促进民族、宗教、对台、外事、侨务、档案、方志、保密等事业发展。

　　（三）统筹城乡发展，走可持续发展之路

　　强化中心城区的综合服务功能。按照科学发展观的要求，高标准做好城市总体规划修编。坚持经营城市理念，加快南城区基础设施建设。继续抓好白云大道改造和城区出口道路、体育文化中心、天然气管网工程、三水厂管网配套、第二垃圾处理场建设。加快东宝山公园建设，抓好东宝山、象山封山育林，增加公用绿地。加强市容环境、交通秩序的综合整治，形成城市现代化管理的长效机制。

　　发展壮大县域经济。落实简政放权政策，支持扩权县市扩大经济社会管理权限，支持东宝区实行比照县市管理体制。学习推广京山发展县域经济经验，培育特色优势产业，壮大骨干财源，增强整体经济实力。积极发展城郊经济和特色城镇经济，抓好宋河、后港、胡集、漳河等中心城镇建设，推进城镇化健康发展。大力加强农村交通、水利、电力、通信等基础设施建设和“一池三改”的农村生态家园建设，改善农村生产生活条件。做好武荆高速、随岳高速荆门段开工和荆宜高速公路建设的服务工作。

　　促进人与自然和谐发展。完善计划生育利益导向机制，继续稳定低生育水平，提高出生人口素质，综合治理出生人口性别比失衡问题。做好全市1％的人口抽样调查工作。保护和合理开发利用土地、水、森林、矿产等资源。认真开展石膏矿、磷矿的综合整顿；继续抓好漳河水源保护，加大农村安全饮水工程建设力度，禁止在水库投肥养鱼；加强工业污染防治，坚决关停“十五小”和“新五小”企业。重视退耕还林工作，加快长江防护林、“绿色通道”建设和湿地保护等重点生态工程建设。积极发展生态示范园区。健全资源有偿使用制度和环保一票否决制。推行清洁生产和资源综合利用，发展循环经济，建设节约型城市。

　　（四）加强社会建设和管理，维护社会稳定

　　推进社区建设。高度重视社区作为社会管理终端与公共服务平台的作用，通过建立新型的社区管理体制，逐步转移政府的社会化职能；通过加强社区基础设施建设，逐步建立数字化社区服务中心和服务站；通过推动社区服务的市场化运作、实体化经营、产业化发展，建设管理有序、功能完备、文明和谐的新型社区。

　　维护社会稳定。制定发展规划、出台政策措施、确定发展项目，要充分考虑人民群众的根本利益，努力从源头上预防和减少不稳定因素。高度重视和正确处理好人民内部矛盾，积极预防和妥善处理群体性事件。认真贯彻实施《信访条例》，健全信访工作责任制，加强人民调解工作，畅通民愿诉求渠道，引导群众以理性方式表达合理要求，切实保障群众的合法权益。坚决纠正征地拆迁、土地流转、企业改制等过程中侵害群众利益的行为。加强物价监测和管理，整顿规范医疗、教育、中介、建设项目等价格和收费秩序。认真做好食品、药品、农资等重要生活生产资料的市场检查检测工作。全面加强社会治安综合治理，深入开展创建“平安荆门”活动，加大对“黄赌毒”查禁力度，依法打击各类刑事犯罪和经济犯罪，严厉打击邪教组织，坚决铲除黑恶势力。建立健全突发事件应急预警机制，提高维护公共安全与社会稳定的能力。高度重视并切实加强劳动保护和安全生产工作，积极排查和消除安全隐患，防止重特大事故发生。

　　五、加强政府行政能力建设

　　坚持科学、民主与依法行政，按照“依法行政、规范管理、权责明晰、纪律严明”的要求，切实提高行政效率，努力降低行政成本和社会成本。

　　牢固树立科学发展的新理念。以科学发展观统揽全局，把发展的定位放到依托特色资源、发挥比较优势、发展特色经济，培植有竞争力的优势产业，提高综合竞争力上来；把发展的重心放到调整经济结构和转变经济增长方式上来；把发展的标准放到以人为本，维护群众利益、保护生态环境、节约自然资源，促进人与自然的和谐发展上来。

　　增强驾驭市场经济的新本领。注重学习和研究市场经济规律，坚持发挥市场对资源配置的基础性作用，始终把握经济工作的主动权。建立健全公众参与、专家论证和政府决定相结合的公开透明的行政决策机制，对关系群众切身利益的重大事项实行公示、听证等制度，推进决策民主化和科学化。做好经济普查工作，科学研究编制“十一五”发展蓝图。努力加快经济管理重心的转变，从注重生产环节向注重市场需求转变，从注重项目投入、经济规模向注重投入、产出、效益相统一转变，从注重生产总值向注重增加财力和城乡居民收入转变，从注重行政手段向注重经济、法律和必要的行政手段相结合转变。

　　塑造法治型、服务型政府的新形象。全面推进依法治市，认真贯彻实施行政许可法，增强依法行政能力。自觉接受人大监督，认真执行人大决议，加强同政协和各民主党派、工商联、无党派人士的民主协商，认真办理人大代表议案、建议和政协委员提案，认真听取工、青、妇等人民团体意见，接受新闻舆论和社会公众监督。各级政府及部门要按照法定权限和程序行使职权，履行职责，做到严格执法、公正执法和文明执法。加强行政执法监督，规范行政执法行为，探索相对集中行政处罚权和综合执法工作。继续推进行政执法责任制和执法过错责任追究制，着力解决职能交叉、多头执法、重复处罚、执法扰民等问题。积极开展法律援助，加强司法、仲裁和行政复议工作。推进基层民主制度建设，做好第六届村委会换届选举工作。正确履行政府职能，在改进经济调节和市场监管的同时，更加强化政府社会管理和公共服务。继续深化行政审批制度改革，以提高行政服务效率为重点，实现“五个中心”良性互动。大力推行政务公开，建立政府新闻发言人制度，加强电子政务建设与应用，推进政府信息的公开与资源共享。努力提高“12345”市长热线服务质量，开设“行风热线”，完善行政投诉机制。

　　创建求真务实、开拓创新的新政风。以保持共产党员先进性教育活动为契机，深入开展“做人民满意公务员”活动。继续加强政风建设，整顿机关作风，健全政府工作责任制，完善目标管理考核体系和办法，加大督查力度，提高政府工作执行力。努力形成领导干部深入调查研究和理性思考的风气，形成讲真话、办实事、求实效的风气，形成勇于创新、狠抓落实的风气。牢记“两个务必”，加大廉政建设和反腐工作力度，充分发挥行政监察、审计等职能部门作用，加强经济责任审计，力行“勤政为民、廉洁高效”。

　　各位代表，为人民服务是人民政府的根本宗旨。今年市政府承诺，以困难群众为重点，办好十件实事：一是提供2.4万个就业岗位，帮助1.5万名下岗失业人员实现再就业。二是建设经济适用住房3万平方米。三是继续加强漳河水源环境保护；提高城区空气质量；解决农村1万人安全饮水问题。四是新建通村公路500公里。五是确保年内村村通电话、通广播电视，新增农村电视用户4万户。六是切实抓好柴湖扶贫项目和漳河库区11个扶贫项目建设；继续在市直机关和县级以上干部中开展结对扶贫济困活动；农村特困人口救助新增6000人。七是积极推进农村新型合作医疗试点工作；建立城乡特困群众医疗救助制度，对晚期血吸虫病人实行免费治疗。八是确保3万名贫困学生完成九年义务教育，改造农村中小学D级危房3.2万平方米。九是建成开放青少年科技馆。十是完成竹皮河城区中段3.7公里河床改造工程。

　　各位代表，荆门正处在经济社会全面快速发展的新阶段，我们深感任务艰巨、责任重大、前途光明。让我们紧密团结在以胡锦涛同志为总书记的党中央周围，在省委、省政府和市委的正确领导下，解放思想，求真务实，改革创新，团结协作，为全面完成今年的各项任务，夺取改革开放和现代化建设的新胜利而努力奋斗！
